# *Brachypodium distachyon* as a model system for studies of copper transport in cereal crops

**DOI:** 10.3389/fpls.2014.00236

**Published:** 2014-05-30

**Authors:** Ha-il Jung, Sheena R. Gayomba, Jiapei Yan, Olena K. Vatamaniuk

**Affiliations:** Department of Crop and Soil Sciences, Cornell UniversityIthaca, NY, USA

**Keywords:** *Brachypodium*, CTR/COPT transporters, copper transport, copper homeostasis, wheat

## Abstract

Copper (Cu) is an essential micronutrient that performs a remarkable array of functions in plants including photosynthesis, cell wall remodeling, flowering, and seed set. Of the world's major cereal crops, wheat, barley, and oat are the most sensitive to Cu deficiency. Cu deficient soils include alkaline soils, which occupy approximately 30% of the world's arable lands, and organic soils that occupy an estimated 19% of arable land in Europe. We used *Brachypodium distachyon* (brachypodium) as a proxy for wheat and other grain cereals to initiate analyses of the molecular mechanisms underlying their increased susceptibility to Cu deficiency. In this report, we focus on members of the CTR/COPT family of Cu transporters because their homologs in *A. thaliana* are transcriptionally upregulated in Cu-limited conditions and are involved either in Cu uptake from soils into epidermal cells in the root, or long-distance transport and distribution of Cu in photosynthetic tissues. We found that of five COPT proteins in brachypodium, BdCOPT3, and BdCOPT4 localize to the plasma membrane and are transcriptionally upregulated in roots and leaves by Cu deficiency. We also found that BdCOPT3, BdCOPT4, and BdCOPT5 confer low affinity Cu transport, in contrast to their counterparts in *A. thaliana* that confer high affinity Cu transport. These data suggest that increased sensitivity to Cu deficiency in some grass species may arise from lower efficiency and, possibly, other properties of components of Cu uptake and tissue partitioning systems and reinforce the importance of using brachypodium as a model for the comprehensive analyses of Cu homeostasis in cereal crops.

## Introduction

Copper (Cu) is an essential micronutrient for all organisms because it acts as a cofactor for enzymes participating in important biological processes such as respiration, photosynthesis, and scavenging of oxidative stress (Burkhead et al., 2009; Merchant, 2010; Ravet and Pilon, 2013). In addition to these functions, plants also employ Cu for the perception of ethylene, nitrogen metabolism, molybdenum cofactor synthesis, cell wall remodeling, response to pathogens, flowering, and seed set (Shorrocks and Alloway, 1988; Marschner, 1995; Burkhead et al., 2009; Mendel and Kruse, 2012; Ravet and Pilon, 2013). This remarkable array of functions is attributed to the ability of Cu to change its oxidation state (Cu^2+^ ↔ Cu^+^). However, the same property imposes toxicity when free Cu ions accumulate in cells in excess because of their ability to promote oxidative stress (Valko et al., 2005). Copper availability, and thus crop productivity on agricultural soils depend on soil type and agricultural practices (Shorrocks and Alloway, 1988; Marschner, 1995; Solberg et al., 1999). For example, Cu deficiency develops on alkaline soils due to low solubility of Cu at high pH, and on organic soils due to Cu binding to organic matter (Shorrocks and Alloway, 1988; Marschner, 1995; Solberg et al., 1999). While Cu deficiency can be remedied by the application of Cu-based fertilizers, this strategy is not environmentally friendly, and the repeated use of fertilizers, as well as Cu-based pesticides, has led to the build-up of toxic levels of Cu in soils (Shorrocks and Alloway, 1988; Marschner, 1995). In this regard, organic farming has emerged as a preferred production system that relies on using natural fertilizers; however natural fertilizers increase soil organic matter and thus, further reduce Cu bioavailability. Sensitivity to Cu bioavailability in soils varies among crops species. Of the major cereal crops, wheat is regarded as the most sensitive to Cu deficiency (Shorrocks and Alloway, 1988; Solberg et al., 1999). In contrast, crops like canola have not shown Cu deficiency symptoms or responded to Cu fertilizer when grown on soils that would cause Cu deficiency in wheat or barley (Solberg et al., 1999). Higher sensitivity of some cereal crops to Cu deficiency compared to other crop species has been recognized in farm reports and fact sheets from some states in the United States and different countries in the world. Among the major cereal crops, sensitivity to Cu deficiency is reported to be in the following rank order, from higher to lower sensitivity: winter wheat > spring wheat > flax > barley > oats > triticale > rye (Shorrocks and Alloway, 1988; Solberg et al., 1999). Remarkably, the molecular mechanisms that underlie this tendency are unknown.

Plants tightly regulate Cu homeostasis to prevent deficiency while avoiding toxicity. This regulation includes transcriptional control of genes encoding proteins that are involved in Cu uptake, trafficking, tissue partitioning, and reallocation among Cu requiring enzymes. Of known Cu transporters, members of the CTR/COPT family are among the main contributors to initial Cu uptake in plants, the green alga *Chlamydomonas reinhardtii*, yeast, *Drosophila*, and humans (Merchant, 2010; Nevitt et al., 2012; Ravet and Pilon, 2013). The plant CTR/COPT family is best characterized in *Arabidopsis thaliana* and *Oryza sativa*, where it is represented by six and seven members, respectively (Peñarrubia et al., 2010; Yuan et al., 2011). *A. thaliana* COPT1, COPT2, and COPT6 are transcriptionally regulated by Cu deficiency, localize to the plasma membrane, mediate Cu uptake, and complement the growth defect of the *S. cerevisiae* Cu uptake mutant lacking functional Cu transporters, Ctr1p, Ctr2p, and Ctr3p (*ctr1*Δ*ctr3*Δ or *ctr1*Δ*ctr2*Δ*ctr3*Δ) (Sancenon et al., 2004; Andres-Colas et al., 2010; Jung et al., 2012; Garcia-Molina et al., 2013; Gayomba et al., 2013; Perea-García et al., 2013). COPT1 and COPT2 function primarily in Cu uptake into the root, while COPT6 also contributes to Cu partitioning in photosynthetic tissues (Jung et al., 2012; Garcia-Molina et al., 2013). In contrast, COPT5 localizes to the tonoplast and the pre-vacuolar compartment and functions by remobilizing Cu from these organelles during Cu deficiency (Garcia-Molina et al., 2011; Klaumann et al., 2011). CTR/COPT proteins homotrimerize to form a pore within the membrane to transport Cu across the lipid bilayer, but can also form heterocomplexes with other CTR/COPT family members and/or other proteins (Lee et al., 2002; De Feo et al., 2009; Yuan et al., 2010, 2011). Studies in *A. thaliana* have shown that while COPT6 interacts with COPT1, this interaction is not required for the ability of COPT6 or COPT1 to complement the Cu uptake deficiency phenotype of the *S. cerevisiae ctr1*Δ*ctr2*Δ*ctr3*Δ mutant strain (Jung et al., 2012). Unlike CTR/COPT transporters from *A. thaliana*, CTR/COPT transporters from *O. sativa* complement the Cu uptake mutant of yeast only when are co-expressed with another OsCOPT member, and OsCOPT7 is the only high-affinity Cu transporter in the *O. sativa* CTR/COPT family (Yuan et al., 2010, 2011). These findings suggest that some properties of CTR/COPT transporters are distinct between grass and non-grass species. These differences are not entirely surprising, given that *A. thaliana* is only distantly related to the *Poaceae* family and lacks many biological features of monocotyledonous grass crops (Brkljacic et al., 2011).

A member of the grass species, *Brachypodium distachyon* (from then on referred to as brachypodium) has emerged as a valuable experimental model for the study of small-grain cereals due to its less complex and fully sequenced genome, the continued development of numerous genetic resources, including efficient protocols for *Agrobacterium*-mediated transformation (Vogel and Hill, 2008), whole genome TILLING mutant alleles (Brkljacic et al., 2011; Thole et al., 2012), a T-DNA insertion mutant collection (http://brachypodium.pw.usda.gov/TDNA/; Bragg et al., 2012), as well as other attributes such as short life cycle (8–12 weeks), small stature (15–20 cm), diploid accessions, self-fertility, and simple growth requirements (Brkljacic et al., 2011). Because brachypodium and wheat grains have similar structure, brachypodium is an attractive model for molecular, genetic, and genomic studies of Cu homeostasis of wheat (Brkljacic et al., 2011; Mochida et al., 2011; Mur et al., 2011). Furthermore, root anatomy of brachypodium and wheat is similar and is distinct from the root anatomy of another *Poaceae* family member, *O. sativa*, which is specialized for overcoming anaerobic conditions associated with submerged roots, and thus, it is suggested that brachypodium and wheat may have similar root-related genes, including those that are involved in mineral ion uptake (Chochois et al., 2012). Hence, brachypodium becomes a preferred model for studies of ion homeostasis, as was recently demonstrated by the analyses of brachypodium Yellow Stripe-Like (YSL) proteins, which are involved in uptake and internal translocation of iron (Fe) (Yordem et al., 2011).

Members of the CTR/COPT family of Cu transporters are the best characterized in both, *A. thaliana* and rice, but corresponding members in brachypodium have not yet been characterized, and are not fully annotated, according to database collections such as the National Center for Biotechnology Information (NCBI, http://www.ncbi.nlm.nih.gov/). In contrast, a specialized database for *A. thaliana* membrane proteins, ARAMEMNON 7.0 (http://aramemnon.botanik.uni-koeln.de/; Schwacke et al., 2003) has a more complete annotation of putative membrane proteins of *A. thaliana* and provides their homologs from other species, including brachypodium, based on amino acid similarity and motif organization. According to *in silico* predictions using ARAMEMNON 7.0, we have identified five putative CTR/COPT family members in brachypodium and provided the initial characterization of their function in Cu homeostasis. We have also developed growth conditions for studies of Cu homeostasis in brachypodium as a model for small grain cereals and established protocols for the isolation and transfection of protoplasts isolated from brachypodium mesophyll cells. We used these procedures, along with functional complementation assays in the *S. cerevisiae ctr1*Δ*ctr2*Δ*ctr3*Δ mutant strain, gene expression analyses, and yeast-two-hybrid assays of protein-protein interactions to study members of the CTR/COPT family of Cu transporters in brachypodium.

## Methods and materials

### Plant material and growth conditions

Seeds of the 21-3 inbred line of brachypodium (Vogel and Hill, 2008) were sterilized in 100% ethanol for 1 min and rinsed three times with sterile water. The lamella and palea were softened by further incubation in sterile water for 2 h and removed with forceps, taking care not to damage the seed. Prepared seeds were then spread onto rinsed perlite irrigated with a standard hydroponic solution, described below. After stratification at 4°C for 24 h, plants were grown for 7 days at 22°C; 12-h light/12-h dark photoperiod at photosynthetic photon flux density of 150 μmol photons m^−2^s^−1^ before transferring to a hydroponic solution prepared as described (Arteca and Arteca, 2000) except that Cu (as CuSO_4_) was added at a higher concentration of 0.25 μM. For Cu limitation and sensitivity assays, 7-day-old seedlings were transferred from perlite to hydroponic solution supplemented with the indicated concentrations of CuSO_4_, or without CuSO_4_ but with the specific Cu chelator, bathocuproine disulfonate (BCS) (Rapisarda et al., 2002). Plants were grown for 18 days before subsequent analyses. For all experiments, the hydroponic solution was changed every 7 days.

### RNA extraction

Root and leaf tissues were separated from 25-day-old plants grown under the indicated conditions and flash-frozen in liquid nitrogen. Samples were homogenized in liquid nitrogen using a mortar and a pestle, and total RNA was isolated using the Plant RNA Kit (Omega Bio-Tek), according to the manufacturer's instructions. Genomic DNA in RNA samples was digested with DNAse *I* (Omega Bio-Tek) prior to cDNA synthesis using the iScript cDNA Synthesis kit (BioRad).

### Quantitative real-time (qRT)-PCR analysis

Prior to qRT-PCR analysis, primers (Supplemental Table 1) and cDNA concentrations were optimized to reach a qRT-PCR amplification efficiency of 100 ± 10%. Two microliters of 10-fold diluted cDNA was used as a template in a total reaction volume of 10 μl containing 500 nM of each PCR primer, 50 mM KCl, 20 mM Tris-HCI, pH 8.4, 0.2 mM dNTPs, and 1.25 units of iTaq DNA polymerase in iQ SYBR Green Supermix (BioRad), containing 3 mM MgCl_2_, SYBR Green I, 20 nM fluoresceine, and stabilizers. PCR was carried out using the CFX96 Real-Time PCR system (BioRad). The thermal cycling parameters were as follows: denaturation at 95°C for 3 min, followed by 39 cycles of 95°C for 10 s then 55°C for 30 s. Amplicon dissociation curves, i.e., melting curves, were recorded after cycle 39 by heating from 60 to 95°C with 0.5°C increments and an average ramp speed of 3.3°C s^−1^. Data were analyzed using the CFX Manager Software, version 1.5 (BioRad). A brachypodium gene encoding ACTIN2 was used as a reference for normalizing gene expression. qRT-PCR experiments were conducted using three independent biological samples, each consisting of three technical replicates, unless indicated otherwise. Statistical analysis was performed using the Relative Expression Software Tool (REST, Qiagen; Pfaffl et al., 2002).

### Plasmid construction

Total RNA isolated from brachypodium leaves and oligonucleotides pairs that are indicated in Supplemental Table 1 were used for RT-PCR amplification of *BdCOPT1*, *BdCOPT3*, *BdCOPT4*, and *BdCOPT5* cDNAs with or without the stop codon. Primers were designed to contain *attB* sites on resulting PCR products for subsequent Gateway cloning (Invitrogen) into the DONR-Zeo entry vector (Invitrogen) and appropriate destination vectors described below.

### Functional complementation of the *S. cerevisiae* copper uptake deficient *ctr1*Δ*ctr2*Δ*ctr3*Δ mutant strain

*S. cerevisiae* SEY6210 (*MATa ura3-52 leu2-3,-112 his3*Δ*200 trp1*Δ*901 lys2-801 suc2*Δ*9*) wild-type and the *ctr1*Δ*ctr2*Δ*ctr3*Δ triple mutant (*MATa ura3-52 his3*Δ*200 trp1-901 ctr1::ura3::Knr ctr2::HIS3 ctr3::TRP1*) used for functional complementation assays were the generous gift of Dr. Dennis Thiele (Duke University). Yeast cells were transformed with YES3-Gate-BdCOPT1, YES3-Gate-BdCOPT3, YES3-Gate-BdCOPT4, YES3-Gate-BdCOPT5 constructs or an empty YES3-Gate vector using the Frozen-EZ yeast Transformation II Kit (Zymo Research). Transformants were selected for uracil prototrophy on YNB medium (YNB-Ura) containing 0.67% (w/v) Yeast Nitrogen Base without amino acids (Difco), 0.077% (w/v) CSM-Ura, 0.05% (w/v) NaCl, 2% dextrose, 2% (w/v) agar. Respiration competence was evaluated by testing the ability of transformants to grow on the non-fermentable carbon sources, glycerol and ethanol (YPEG) as described (Dancis et al., 1994a; Puig et al., 2002). Briefly, transformants were grown in liquid YNB-Ura to an OD_600 nm_ = 1.0, serially-10-fold diluted and spotted onto YPEG medium containing 1% (w/v) yeast extract, 2% (w/v) bacto-peptone, 3% (v/v) glycerol 2% (v/v) ethanol, and 2% (w/v) agar and the indicated concentration of CuSO_4_ or onto YNB-Ura for controls. Plates were incubated for 3 days at 30°C.

### Isolation of protoplasts from brachypodium leaves

Protoplasts were isolated from leaves of 25-day-old brachypodium grown as described above. The protoplast isolation procedure was based on (Zhai et al., 2009). Briefly, 0.2 g of young leaf tissue was immersed in 15 ml of filter-sterilized TVL solution (Supplemental Table 2), finely chopped with a fresh razor blade and transferred to a 200 ml beaker containing 20 ml of Enzyme solution (Supplemental Table 2). The beaker was wrapped in aluminum foil to protect samples from light and samples were vacuum infiltrated for 30 min before incubation at 30°C for 60 min. The mixture was then agitated at 35 rpm at room temperature for 18–20 h. Released protoplasts were collected into 50-ml Falcon centrifuge tubes by carefully sieving the mixture through eight layers of cheesecloth, pre-wetted with W5 solution (Supplemental Table 2). To increase the protoplast yield, the cheesecloth was rinsed with an additional 10 ml of W5 solution. Sieved protoplasts were then carefully overlaid with 5 ml of W5 solution and left at room temperature for 1 h to allow protoplasts to float to the interface of Enzyme solution and W5 solution. Fifteen milliliters of protoplasts were collected from the interface and transferred into a new 50 ml Falcon tube containing 20 ml of W5 solution. Protoplasts were collected by centrifugation for 7 min at 100 × *g*. The residual Enzyme solution was removed by two rounds of rinsing protoplasts with 10 ml W5 solution and centrifuged for 5 min at 60 × *g*. Purified protoplasts were resuspended in 3–5 ml W5 solution and the protoplast yield was evaluated by cell counting using a hemocytometer. Protoplast viability was evaluated using the Plant Cell Viability Assay Kit (Sigma), according to manufacturer's recommendations.

### Transfection of protoplasts with plasmid DNA

Protoplasts were transfected using a procedure adopted from (Jung et al., 2011). Briefly, purified protoplasts were incubated on ice for 30 min and centrifuged for 5 min at 60 × *g* and W5 solution was removed. Protoplasts were then re-suspended in MMG solution (Supplemental Table 2) and 100 μl aliquots were transferred into a 2-ml round-bottom microcentrifuge tube. Plasmid DNA (5–10 μg) was added to protoplasts and mixed gently. For controls, DNA was omitted and replaced with equivalent volumes of sterile water (mock transfection). Transfection was initiated by the addition of 110 μl of PEG-calcium solution (Supplemental Table 2). Protoplasts were gently mixed with PEG-calcium solution by tapping the tube followed by incubation for 7 min at room temperature. Transfection was terminated by diluting the mixture by an addition of 700 μl of W5 solution. Transfected protoplasts were collected by centrifugation for 2 min at 100 × *g*, and supernatant was removed to leave 50–100 μl of protoplast suspension. Each sample was then brought up to a volume of 1 ml with W5 solution and incubated in the dark at room temperature for 18 h.

### Subcellular localization and fluorescent microscopy

For studies of the subcellular localization in brachypodium protoplasts, *BdCOPT3*, and *BdCOPT4* cDNAs lacking the stop codon were fused at the C-terminus to the enhanced green fluorescent protein (EGFP) of the SAT6-N1-EGFP-Gate vector and expressed under the control of the cauliflower mosaic virus (CaMV) *35S* promoter. The resulting *35S_pro_-BdCOPT-EGFP* constructs or SAT6-N1-EGFP-Gate lacking cDNA inserts were transfected into brachypodium protoplasts as described above. Plasma membranes were stained with 50 μM FM4-64 as described (Ueda et al., 2001).

For studies of subcellular localization in *S. cerevisiae*, entry clones containing *BdCOPT1*, *BdCOPT3*, *BdCOPT4*, or *BdCOPT5* cDNAs without the stop codon were fused at C-terminus with EGFP in the YES3-EGFP-Gate vector (Jung et al., 2012). The resulting constructs and the empty YES3-EGFP-Gate vector were transformed into *S. cerevisiae ctr1*Δ*ctr2*Δ*ctr3*Δ triple mutant using the Frozen-EZ yeast Transformation II Kit (Zymo Research) and transformants were selected on YNB-Ura medium, as described above. Subcellular localization was assessed in cells grown overnight in liquid YNB-Ura.

EGFP- and FM4-64- mediated fluorescence, and chlorophyll autofluorescence were visualized using FITC (for EGFP) or rhodamine (FM4-64 and chlorophyll) filter sets of the Axio Imager M2 microscope equipped with the motorized Z-drive (Zeiss). Z-stack (1.3 μm-thick) images were collected with the high-resolution AxioCam MR Camera and then 3D deconvoluted using an inverse filter algorithm of the Zeiss AxioVision 4.8 software. Images were processed using the Adobe Photoshop software package, version 12.0.

### Split-ubiquitin membrane yeast two-hybrid system (MYTH)

Vectors and *S. cerevisiae* strains THY.AP4 (*MATa leu2-3,112 ura3-52 trp1-289 lexA::HIS3 lexA::ADE2 lexA::lacZ*) and THY.AP5 (*MAT*α *URA3 leu2-3,112 trp1-289 his3*-Δ*1 ade2*Δ*::loxP*) for MYTH were obtained from the Frommer lab (Stanford University) depository at *Arabidopsis* Biological Resource Center (ABRC) http://www.arabidopsis.org/abrc/index.jsp. AtCOPT6-Cub-PLV and AtCOPT6-NubG fusions were previously described (Jung et al., 2012). Full-length *BdCOPT1, BdCOPT3, BdCOPT4*, and *BdCOPT5* cDNAs without stop codons were introduced into the MetYC-dest and pXN-dest22-3HA destination vectors by Gateway cloning (Invitrogen) to generate bait, BdCOPT-CubPLV, and prey, BdCOPT-NubG, constructs in THY.AP4 and THY.AP5 strains, respectively. In all cases, C- and N terminal fragments of ubiquitin were placed at the C-terminus of the BdCOPT proteins. Protein interactions were selected in diploid cells after 2 days of growth on SC medium lacking adenine and histidine. Interactions were verified using β-galactosidase assays, as detailed in Kittanakom et al. (2009).

### Construction of phylogenetic tree

The phylogenetic tree was built using the Neighbor-Joining method (Saitou and Nei, 1987). The bootstrap consensus tree inferred from 500 replicates is taken to represent the evolutionary history of the taxa analyzed (Felsenstein, 1985). Branches corresponding to partitions reproduced in less than 50% bootstrap replicates are collapsed. The percentage of replicate trees in which the associated taxa clustered together in the bootstrap test (500 replicates) are shown next to the branches (Felsenstein, 1985). The tree is drawn to scale, with branch lengths in the same units as those of the evolutionary distances used to infer the phylogenetic tree. The evolutionary distances were computed using the number of differences method (Nei and Kumar, 2000) and are in units of the number of amino acid differences per sequence. The analysis involved 22 amino acid sequences. All positions containing gaps and missing data were eliminated. There were a total of 75 positions in the final dataset. Evolutionary analyses were conducted in MEGA5 (Tamura et al., 2011) with *A. thaliana* IRT1 as an outgroup.

### Accession numbers

Accession numbers for genes used in this study were according to nomenclature from ARAMEMNON 7.0 (http://aramemnon.botanik.uni-koeln.de/) and MIPS (http://mips.helmholtz-muenchen.de/plant/brachypodium/): *Bradi1g24180* (*BdCOPT1*), *Bradi1g24190* (*BdCOPT2*), *Bradi2g51210* (*BdCOPT3*), *Bradi4g31330* (*BdCOPT4*), *Bradi5g09580* (*BdCOPT5), Bradi1g10630.1* (*BdACTIN*), and *At2g26975* (*AtCOPT6*).

## Results

### The predicted COPT transporters of brachypodium share conserved features of the CTR/COPT family

**Figure 1:**
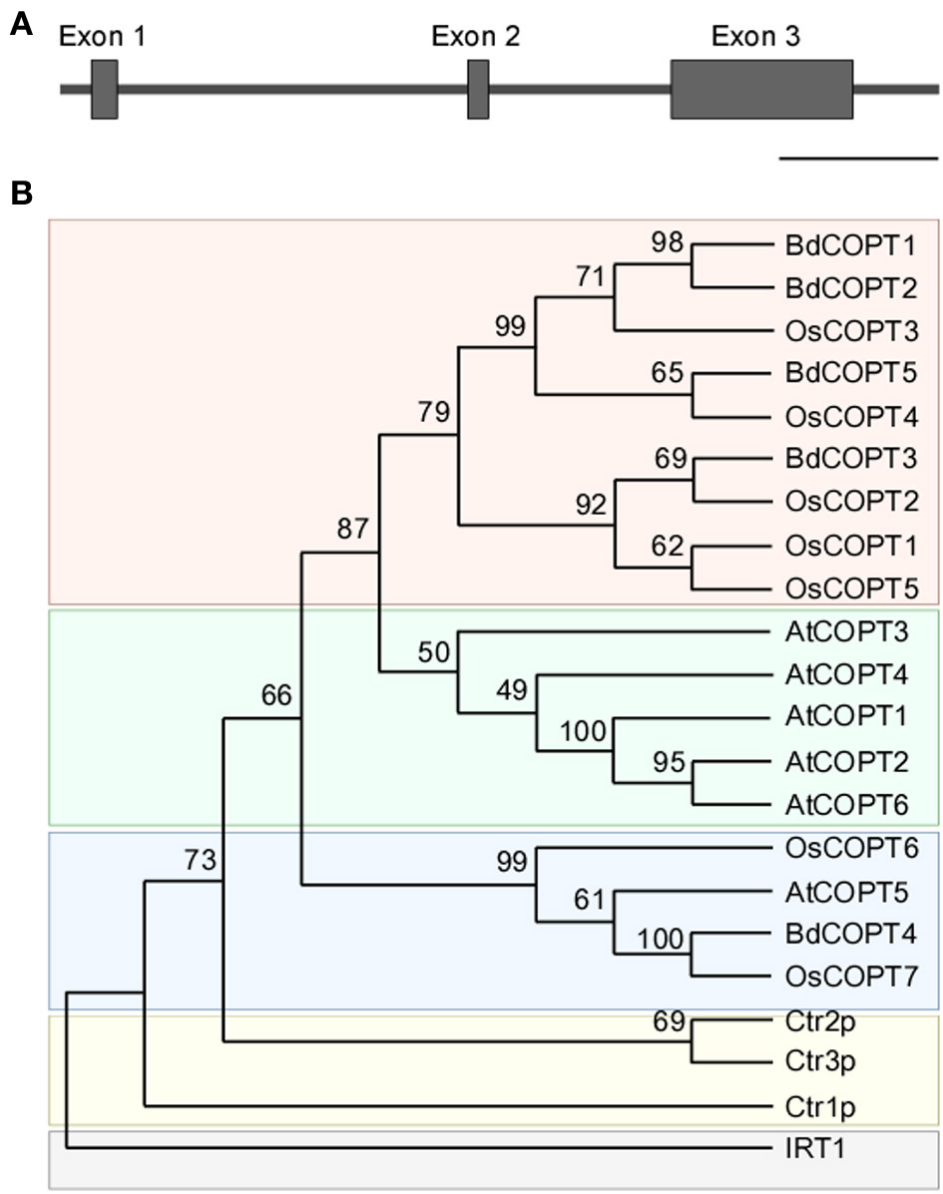
**Gene structure of *BdCOPT2* and phylogenetic analysis of the brachypodium COPT family. (A)** Gene structure of *BdCOPT2*. Note the presence of introns, which are absent in other plant CTR/COPT members. Scale bar = 500 bp. **(B)** A phylogenetic tree of CTR/COPT members of *A. thaliana* (designated as AtCOPT1-6), *O. sativa* (designated as OsCOPT1-7), brachypodium (designated as BdCOPT1-5), and *S. cerevisiae* (designated as Ctr1p-CTR3p). The *A. thaliana* protein, IRT1, is included as an outgroup.

To identify putative members of the CTR/COPT family in brachypodium, we used the Plant Membrane Protein database, ARAMEMNON 7.0 (http://aramemnon.botanik.uni-koeln.de/; Schwacke et al., 2003), which has the most complete annotation of putative CTR/COPT transporters based on amino acid similarity and motif organization of CTR/COPT proteins in different species. We found that the brachypodium genome possesses five genes that encode putative CTR/COPT transporters *Bradi1g24180*, *Bradi1g24190*, *Bradi2g51210*, *Bradi4g31330*, *Bradi5g09580*, designated *COPT1* through *COPT5* (*alias BdCOPT1*-*BdCOPT5*) (Table 1). *BdCOPT1* and *BdCOPT2* are located on chromosome 1, while *BdCOPT3*, *BdCOPT4*, and *BdCOPT5* are found on chromosome 2, 4, and 5, respectively (Table 1). Based on the *BdCOPT* gene structures in the ARAMEMNON 7.0 database, *BdCOPT1*, *BdCOPT3, BdCOPT4*, and *BdCOPT5* lack introns, similar to *COPT* genes in rice (Yuan et al., 2011) and *A. thaliana* (http://arabidopsis.org). In contrast, *BdCOPT2* possesses two introns (Figure 1A).

**Table 1:** **The proposed nomenclature and accession numbers of putative brachypodium COPT transporters annotated at different databases**.

**Suggested nomenclature**	**MIPS/ARAMEMNON**	**NCBI**	**UniPROT**	**Chromosome**
*BdCOPT1*	*Bradi1g24180*	*not annotated*	*I1GT99*	I
*BdCOPT2*	*Bradi1g24190*	*not annotated*	*I1GTA0*	I
*BdCOPT3*	*Bradi2g51210*	*XP_003569917.1*	*I1HS28*	II
*BdCOPT4*	*Bradi4g31330*	*XP_003578182.1*	*I1IQG8*	IV
*BdCOPT5*	*Bradi5g09580*	*not annotated*	*I1IXK4*	V

BdCOPTs share 16–69% amino acid sequence identity and 26–73% sequence similarity to each other (Table 2). Phylogenetic analysis revealed that a majority of CTR/COPT proteins from brachypodium and *O. sativa* cluster together and are separate from CTR/COPTs from *A. thaliana* (Figure 1B). One exception is BdCOPT4, which forms a separate cluster along with AtCOPT5, OsCOPT7, and OsCOPT6 (Figure 1B). BdCOPT4 is highly similar (65%) to AtCOPT5, whereas other BdCOPT proteins share 38–54% similarity to their counterparts in *A. thaliana* (Table 3). Similar to *OsCOPT* members (Yuan et al., 2011), the nucleotide sequence of *BdCOPT* genes is GC-rich, ranging from GC content of 67.9% (*BdCOPT2*) to 73.2% (*BdCOPT3*), in contrast to an average GC content of 50% in *A. thaliana COPT*s.

**Table 2:** **Percentage of amino acid identity (similarity) among putative COPT proteins in brachypodium**.

	**BdCOPT1**	**BdCOPT2**	**BdCOPT3**	**BdCOPT4**	**BdCOPT5**
BdCOPT1	100 (100)	69 (73)	40 (48)	21 (30)	43 (51)
BdCOPT2	–	100 (100)	34 (42)	16 (26)	38 (46)
BdCOPT3	–	–	100 (100)	27 (35)	42 (50)
BdCOPT4	–	–	–	100 (100)	31 (42)
BdCOPT5	–	–	–	–	100 (100)

**Table 3:** **Amino acid length (aa length), molecular mass (shown in kDa), and percentage of amino acid identity (similarity) of putative brachypodium COPT (BdCOPT1-5) transporters in comparison to their homologs in *A. thaliana* (AtCOPT1-6)**.

	**aa length/kDa**	**AtCOPT1**	**AtCOPT2**	**AtCOPT3**	**AtCOPT4**	**AtCOPT5**	**AtCOPT6**
BdCOPT1	183/18.8	40.0 (47.1)	36.7 (47.5)	38.4 (49.0)	34.5 (43.4)	28.1 (41.8)	42.8 (54.5)
BdCOPT2	214/22.5	35.9 (44.7)	34.2 (45.6)	36.4 (47.0)	33.1 (46.2)	26.0 (38.4)	41.4 (53.8)
BdCOPT3	162/16.6	41.4 (53.7)	40.5 (53.8)	36.4 (48.3)	34.5 (48.3)	30.8 (40.4)	43.4 (53.8)
BdCOPT4	151/15.8	31.1 (45.0)	29.8 (44.4)	29.1 (42.4)	26.9 (38.6)	57.5 (65.1)	31.0 (44.1)
BdCOPT5	159/16.3	42.1 (51.6)	38.0 (46.2)	39.7 (51.0)	34.5 (45.5)	28.1 (39.7)	40.7 (52.4)

CTR/COPT family members possess conserved structural features that include three putative transmembrane helices (TMs), the N- and C-termini located toward the extracellular space and cytosol respectively, N-terminally-located methionine-rich motifs (Mets motifs), and M*XXX*M*-X*_12_-G*XXX*G motifs located within TM2 and TM3, respectively (Puig et al., 2002; De Feo et al., 2007, 2009; Peñarrubia et al., 2010). Importantly, M*XXX*M motifs of TM2 in Ctr1p of *S. cerevisiae*, and COPT2 and COPT6 of *A. thaliana* contain a positionally conserved Met residue that is essential for the ability of these proteins to transport Cu (Puig et al., 2002; Jung et al., 2012; Gayomba et al., 2013). Some CTR/COPT proteins also contain the C-terminal cysteine-rich C*X*C motif, which is suggested to bind Cu ions for transfer to cytosolic Cu chaperones, or to downregulate Ctr1p activity in response to toxic Cu levels (Puig et al., 2002; Xiao et al., 2004; Wu et al., 2009). This motif is present in *A. thaliana* COPT1 and COPT2, but is absent in COPT6; nevertheless, COPT6 is a functional Cu transporter (Jung et al., 2012).

**Figure 2:**
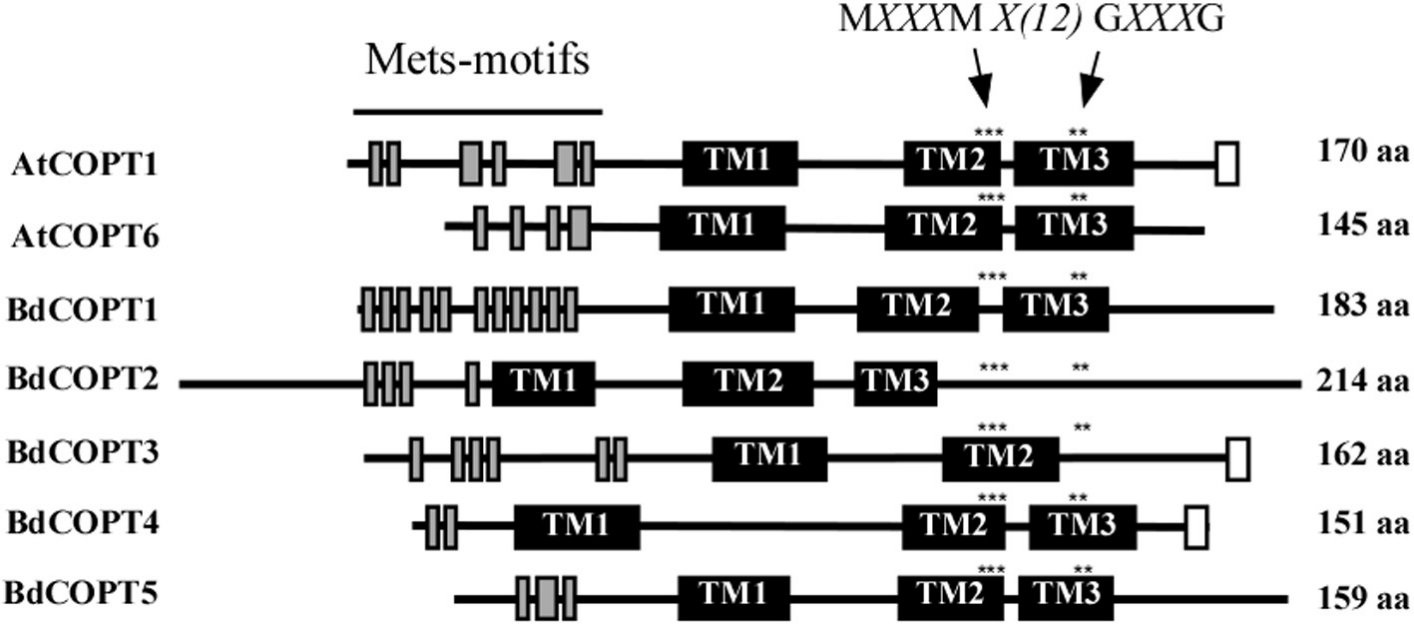
**A diagram showing conserved motifs within the primary sequence of BdCOPT proteins and their homologs in *A. thaliana*, AtCOPT1 and AtCOPT6**. Topology predictions are based on the TMHMM software, version 1.0. Shown are methionine-rich motifs within the predicted N-terminal domain (Mets-motifs, gray bars), transmembrane domains (TM1, TM2, and TM3; black bars) and cysteine-rich motifs within the predicted C-terminal domain (white bars). Asterisks “^***^” and “^**^” indicate the location of conserved M*XXX*M and G*XXX*G motifs. Note that BdCOPT3 has a unique structure of two transmembrane domains and that BdCOPT3 and BdCOPT4 have cysteine-rich domains in the C-terminal domain.

Computer algorithm-assisted analysis of membrane topology and motif organization in brachypodium CTR/COPT proteins revealed that all BdCOPTs, except for BdCOPT3, are predicted to contain the classical three TMs (Figure 2) with N-terminal domains oriented toward the extracellular space, while C-terminal domains are predicted to be located in the cytosol. In contrast, BdCOPT3 is predicted to have two transmembrane domains, with both N- and C-termini located in the cytosol. While polypeptides of all BdCOPT included the N-terminal Mets motifs, the distribution of M*XXX*M*-X*_12_-G*XXX*G motifs varied within different BdCOPT polypeptides (Figure 2). For example, M*XXX*M motifs and thus, positionally conserved essential Met residues, were located within TM2 in BdCOPT3, BdCOPT4, and BdCOPT5, but outside of TM2 in BdCOPT1 and BdCOPT2. Although the predicted membrane topology of BdCOPT proteins has to be validated experimentally, the location of essential positionally conserved Met residues of the M*XXX*M motif outside of the predicted membrane domain suggest that BdCOPT1 and BdCOPT2 might not mediate Cu transport. The C-terminal C*X*C motif is present in BdCOPT3 as a CC motif and in BdCOPT4 as a C*X*C motif, but is absent in BdCOPT1, BdCOPT2, and BdCOPT5 (Figure 2).

### BdCOPT3 and BdCOPT4 respond transcriptionally to Cu status

To determine whether *BdCOPT* genes respond transcriptionally to Cu status of the plant like their counterparts in *A. thaliana* and *O. sativa*, we first developed growth conditions under which Cu limitation would minimally affect the growth and development of brachypodium. Seeds were germinated in perlite irrigated with a modified hydroponic medium supplemented with 0.25 μM CuSO_4_ (Supplemental Figure 1 and Methods and Materials) and grown for 7 days before transferring to hydroponic medium to grow further for 18 days under the following conditions: (1) control conditions (0.25 μM CuSO_4_); (2) Cu limited conditions (0 μM CuSO_4_); (3) Cu deficiency (0 μM CuSO_4_ and supplemented with 500 μM of the Cu chelator, bathocuprione disulfonate (BCS); and (4) Cu excess (3 μM CuSO_4_). While there were no signs of chlorosis in leaves of plants grown under Cu deficiency or Cu excess (Supplemental Figure 2A), plants grown under Cu deficiency had decreased height, and shoot and root dry weight compared to plants grown under control conditions (Supplemental Figure 2B). Plants grown under Cu excess had decreased shoot and root dry weight, but were of the same height as control plants. Cu limitation did not significantly alter plant growth or shoot and root biomass (Supplemental Figure 2B).

**Figure 3:**
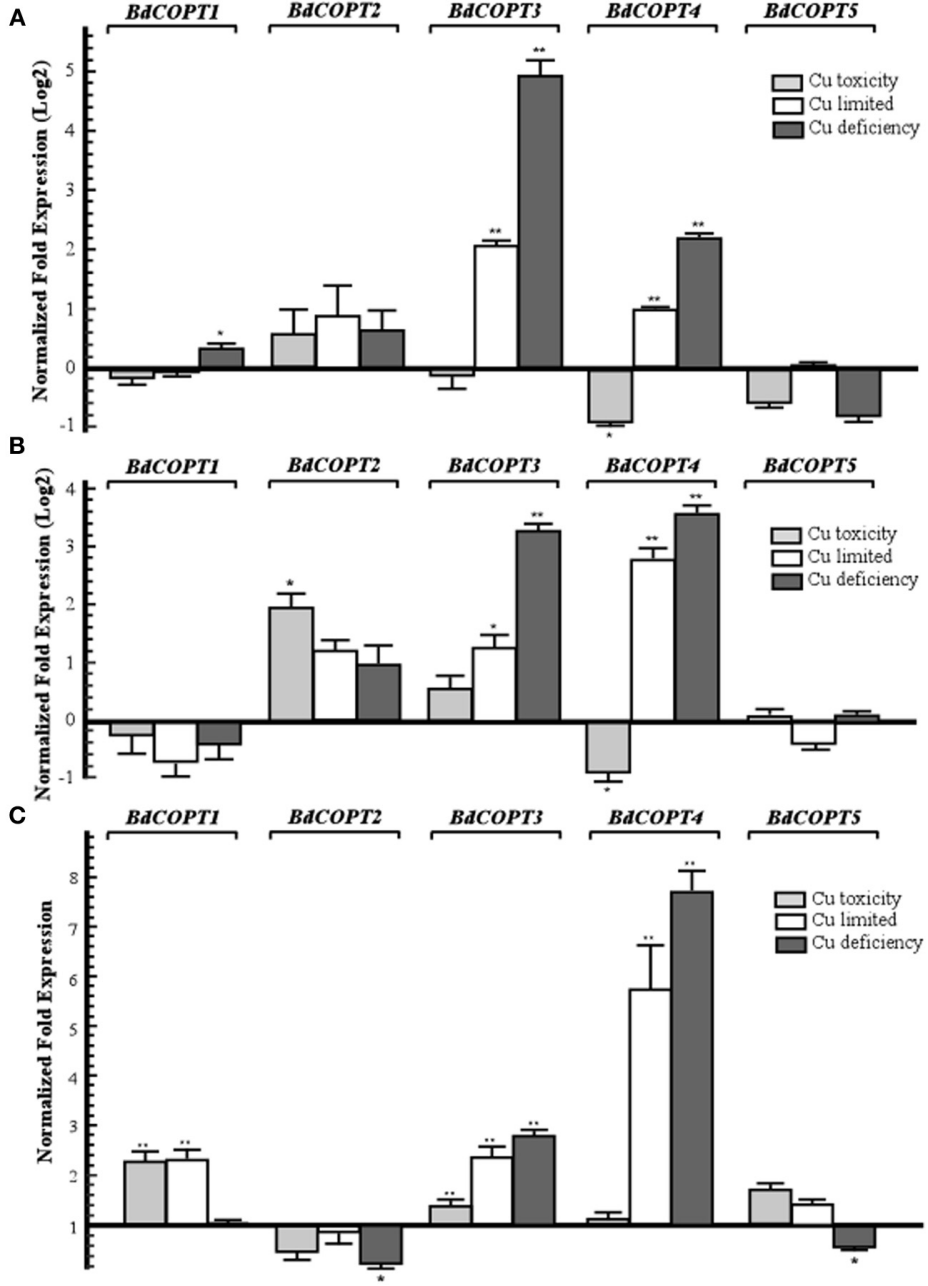
**Quantitative real-time (qRT)-PCR analysis of the effect of Cu on expression *COPT1* through *5* in roots (A), young leaves (B), and older leaves (C) of brachypodium**. For all treatments, 7-day-old wild-type seedlings were transferred to hydroponic solution and were grown for 18 days in medium that, in addition to macro- and micronutrients, contained either 0.25 μM CuSO_4_ (control conditions), 0 μM CuSO_4_ (Cu limited), 0 μM CuSO_4_ + 500 μM BCS (Cu deficiency), or 3 μM CuSO_4_ (Cu toxicity). Error bars show SE (*n* = 9). Differences of the mean values between control and treated plants are indicated as ^*^p ≤ 0.05 or ^**^p ≤ 0.001. Results are presented relative to the expression of genes under control conditions.

We then analyzed the steady-state levels of *BdCOPT* mRNAs in different plant tissues of brachypodium grown under the Cu regimes described above. Expression studies in roots (Figure 3A) revealed that Cu limitation increased mRNA expression of *BdCOPT3* and *BdCOPT4*. Cu deficiency increased *BdCOPT3* and *BdCOPT4* expression even further and, in addition, increased expression of *BdCOPT1*. There were no statistically significant differences in mRNA expression levels of *BdCOPT2* and *BdCOPT5* in root tissue of plants grown under these conditions (Figure 3A). In roots of plants grown under Cu toxicity, *BdCOPT4* was the only gene whose expression was responsive to this treatment (Figure 3A). In young leaves (Figure 3B), Cu limitation elevated the abundance of *BdCOPT3* and *BdCOPT4* transcripts, which increased even further under Cu deficiency. In young leaves of plants grown under Cu toxicity, expression of *BdCOPT4* decreased, but expression of *BdCOPT2* increased. Expression studies in old leaves (Figure 3C) of plants grown in Cu limited or Cu deficient conditions showed an increase of the abundance of *BdCOPT3* and *BdCOPT4* transcripts. In contrast, both, Cu toxic and Cu limited conditions, increased expression of *BdCOPT1*, while expression of *BdCOPT2* and *BdCOPT5* were downregulated under Cu deficiency.

To summarize, of five *BdCOPT* genes, *BdCOPT3*, and *BdCOPT4*, are the most responsive to fluctuations in Cu availability, and changes in their expression are most prominent in roots and young leaves. *BdCOPT2* is significantly upregulated under Cu toxicity only in young leaves, while *BdCOPT1* is regulated by Cu mainly in old leaves. Expression of *BdCOPT5* was the least responsive to Cu availability and was downregulated only by Cu deficiency and only in old leaves.

### Heterologously expressed *BdCOPT3, BdCOPT4*, and *BdCOPT5* partially rescue growth defects of the *S. cerevisiae ctr1*Δ*ctr2*Δ*ctr3*ΔCu uptake mutant on non-fermentable growth medium

We next tested whether BdCOPT proteins are involved in Cu transport. In this regard, the *S. cerevisiae ctr1*Δ*ctr2*Δ*ctr3*Δ mutant, which lacks high-affinity plasma membrane-localized Cu uptake transporters Ctr1p and Ctr3p, and the vacuolar membrane-localized Ctr2p, is deficient in Cu uptake and release of Cu from the vacuole, and has been successfully used to identify Cu transport capabilities of CTR/COPT transporters from higher plants (Kampfenkel et al., 1995; Barhoom et al., 2008; Yuan et al., 2010, 2011; Garcia-Molina et al., 2011; Klaumann et al., 2011; Jung et al., 2012; Gayomba et al., 2013). Analyses of Cu transport capabilities using yeast as a heterologous system is based on the fact that cells lacking functional Cu uptake systems are unable to deliver Cu to cytochrome *c* oxidase in the mitochondrial respiratory chain, preventing cell growth on non-fermentable carbon sources such as glycerol and ethanol (YPEG medium), unless high concentrations of exogenous Cu are added to the growth medium (Dancis et al., 1994b; Glerum et al., 1996). Therefore, we expected that if BdCOPT proteins act as high-affinity Cu transporters, then their expression in the *S. cerevisiae ctr1*Δ*ctr2*Δ*ctr3*Δ mutant would promote Cu uptake and suppress growth defects of the mutant on non-fermentable medium.

**Figure 4:**
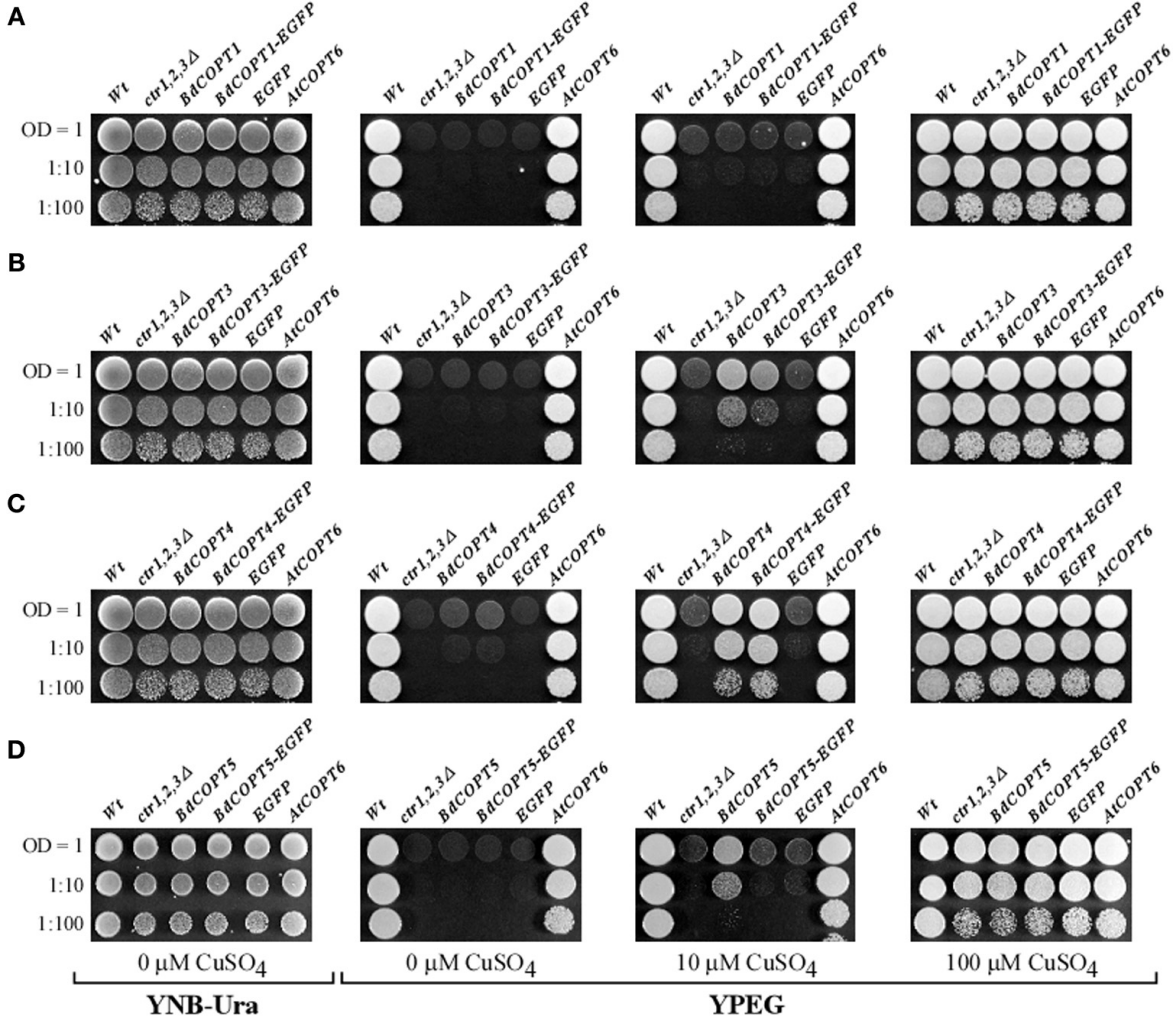
***BdCOPT3*, *BdCOPT4*, and *BdCOPT5* rescue the growth defect of the *S. cerevisiae ctr1*Δ*ctr2*Δ*ctr3*Δ triple mutant on ethanol/glycerol medium (YPEG)**. The *ctr1*Δ*ctr2*Δ*ctr3*Δ mutant was transformed with the YES3-Gate vector harboring *BdCOPT1* **(A)**, *BdCOPT3* **(B)**, *BdCOPT4* **(C)**, and *BdCOPT5* **(D)** along with corresponding EGFP-fusions and spotted onto YPEG plates supplemented with the indicated concentrations of CuSO_4_. As negative controls, the *ctr1*Δ*ctr2*Δ*ctr3*Δ mutant strain was transformed with the empty YES3-Gate (*ctr1,2,3*Δ) or empty YES3-EGFP-Gate vector (*EGFP*). The isogenic wild-type, SEY6210, transformed with the empty YES3-Gate vector (*Wt*) and *ctr1*Δ*ctr2*Δ*ctr3*Δ cells transformed with YES3-Gate harboring the *A. thaliana COPT6* (*AtCOPT6*) cDNA insert were used as positive controls.

Here, we focused on analyses of BdCOPT1, BdCOPT3, BdCOPT4, and BdCOPT5 due to difficulty in cloning of BdCOPT2. As a positive control, we used the previously characterized CTR/COPT transporter from *A. thaliana*, AtCOPT6 (Jung et al., 2012). We found that all yeast lines grew at the same rate on standard medium (YNB-Ura) supplied with glucose as a carbon source or on YPEG medium supplemented with 100 μM CuSO_4_ (Figure 4). In contrast, only the empty vector-expressing wild-type cells and *ctr1*Δ*ctr2*Δ*ctr3*Δ cells expressing *AtCOPT6* grew well on YPEG medium (Figure 4), suggesting that none of BdCOPTs tested were able to rescue the growth defect of the *S. cerevisiae* mutant on YPEG medium. However, addition of a low concentration of Cu (10 μM CuSO_4_) to YPEG medium allowed *BdCOPT3* (Figure 4B), *BdCOPT4* (Figure 4C), and *BdCOPT5* (Figure 4D) but not *BdCOPT1* (Figure 4A) to complement partially the growth defect of *ctr1*Δ*ctr2*Δ*ctr3*Δ cells. These results suggest that transport capabilities of brachypodium COPT transporters differ from their counterparts in *A. thaliana*.

### BdCOPT3 and BdCOPT4 localize to the plasma membrane in *S. cerevisiae* cells and brachypodium protoplasts

*S. cerevisiae* Ctr1p and Ctr3p localize to the plasma membrane and contribute to Cu uptake into the cell (Dancis et al., 1994a; Peña et al., 2000), while Ctr2p localizes to the vacuolar membrane and remobilizes Cu from this internal store upon Cu deficiency (Rees et al., 2004). To determine whether BdCOPTs rescue growth defects of the *S. cerevisiae ctr1*Δ*ctr2*Δ*ctr3*Δ mutant by facilitating Cu uptake into the cell from the external medium or by vacuolar remobilization, we determined their subcellular localization in yeast cells as well as in brachypodium protoplasts.

**Figure 5:**
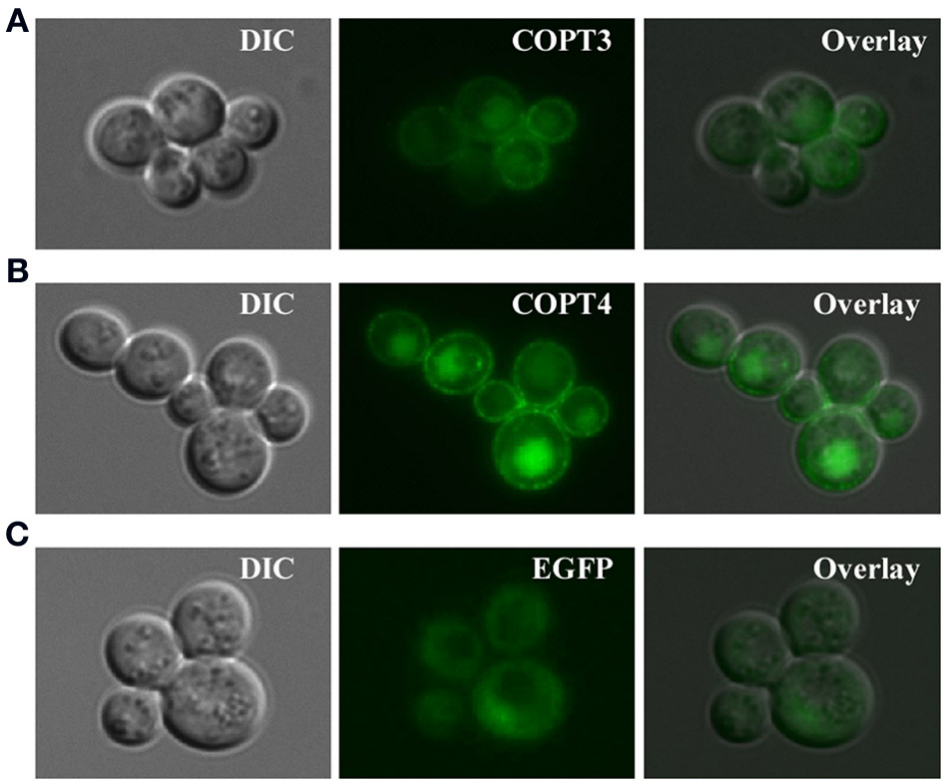
**Subcellular localization of BdCOPT3-EGFP (A) or BdCOPT4-EGFP (B) fusions or EGFP (C) in *S. cerevisiae ctr1*Δ*ctr2*Δ*ctr3*Δ cells**. Superimposed images (Overlay) from differential interference contrast microscopy (DIC) and EGFP-mediated fluorescence (EGFP) show that BdCOPT3 and BdCOPT4 localize to the plasma membrane and that the pattern of fluorescence of EGFP-fused proteins is distinct from EGFP.

For studies in *S. cerevisiae*, we inserted *BdCOPT3*, *BdCOPT4*, and *BdCOPT5* cDNAs without the stop codon into the YES3-EGFP-Gate vector to generate translational C-terminal EGFP fusions. We then verified whether the BdCOPT-EGFP constructs were functional by expressing them in the *ctr1*Δ*ctr2*Δ*ctr3*Δ mutant and assessing the growth of transformed cells on YPEG media. We found that expression of BdCOPT3-, and BdCOPT4-EGFP fusions in *ctr1*Δ*ctr2*Δ*ctr3*Δ cells resulted in growth phenotypes mirroring results of cells expressing un-tagged proteins (Figures 4B,C). However, the BdCOPT5-EGFP construct was unable to rescue growth of *ctr1*Δ*ctr2*Δ*ctr3*Δ cells, suggesting that fusing EGFP with BdCOPT5 resulted in the loss of its activity (Figure 4D). Since unfunctional BdCOPT5-EGFP might mislocalize in yeast cells as well as in brachypodium protoplasts, this construct was omitted from subsequent study. We did not analyze the subcellular localization of BdCOPT1 for the same reason. Fluorescent microscopy revealed that EGFP-mediated fluorescence in BdCOPT3-EGFP and BdCOPT4-EGFP expressing cells localized mainly to the cell periphery and the distribution of the EGFP signal was distinct in *S. cerevisiae* cells expressing the EGFP-only vector (Figure 5). These data suggest that BdCOPT3 and BdCOPT4 localize to the plasma membrane in this heterologous system.

**Figure 6:**
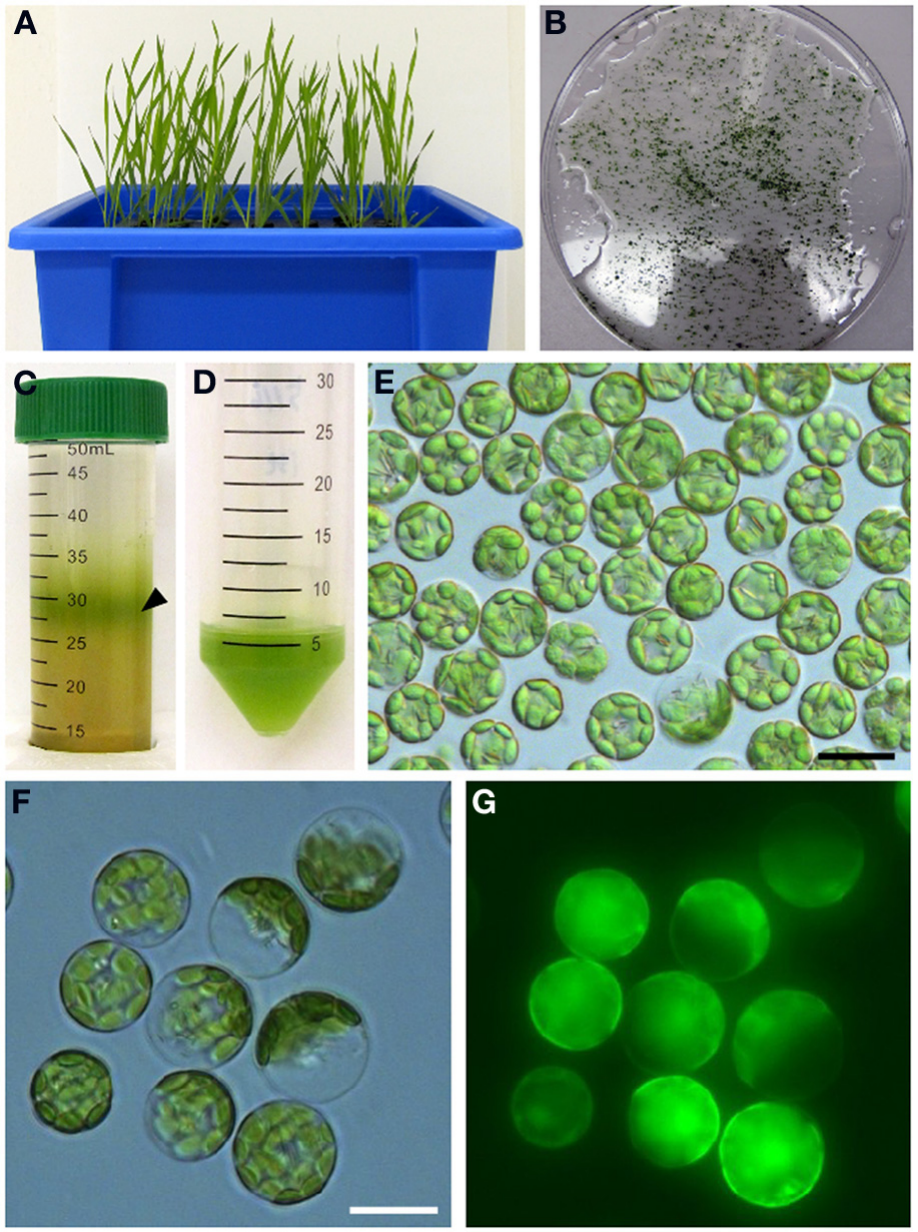
**Isolation of protoplasts from brachypodium**. Hydroponically grown 25-day-old plants **(A)** were used for the isolation of protoplasts from leaf tissue. Chopped brachypodium leaves in filter-sterilized TVL solution are shown in **(B)**. Enzymatic digestion of the cell wall and fractionation by sucrose density gradient yielded protoplasts at the interface of the enzyme solution and W5 solution (**C**, black arrow). Protoplasts were collected and purified from sucrose density gradient solution **(D)** and visualized under microscopy using bright-field filter sets **(E)**. In our method, 0.2 g of leaf tissue from 25-day-old seedlings yields 5 × 10^6^–10^7^ protoplasts. Close-up of brachypodium protoplasts through bright-field **(F)** and FITC **(G)** filter sets to assess protoplast viability after staining with the membrane-permeable non-fluorescent dye, fluorescein diacetate. After diffusion into viable protoplasts fluorescein diacetate is hydrolyzed into a polar compound, causing the cytosoplasm of the cell to fluoresce under the FITC filter set. Scale bar = 20 μm.

**Figure 7:**
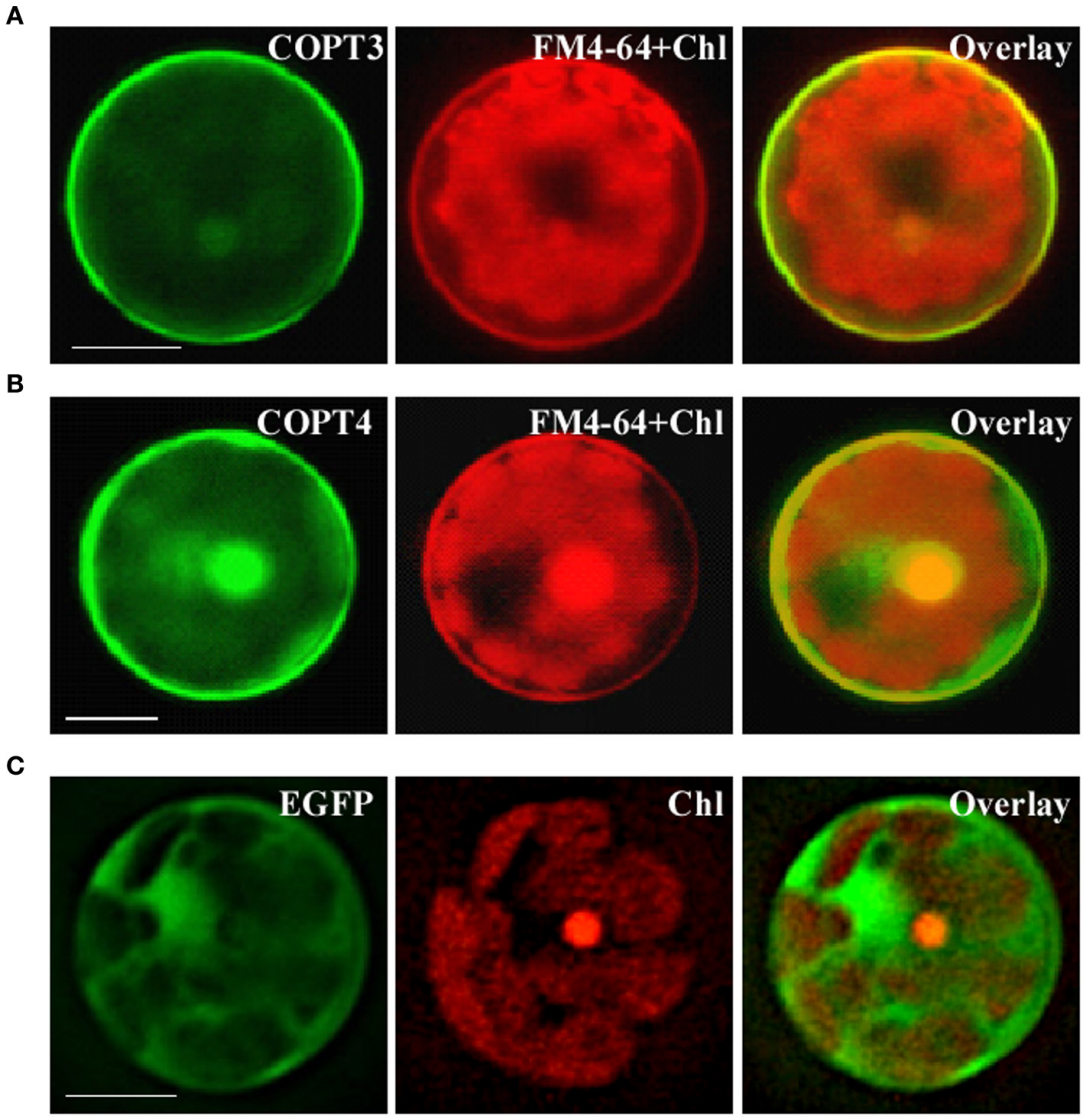
**Subcellular localization of BdCOPT3 and BdCOPT4 in brachypodium protoplasts**. Protoplasts isolated from leaves of 25-day-old plants were transfected with BdCOPT3-EGFP **(A)** or BdCOPT4-EGFP **(B)** constructs or the empty SAT6-N1-EGFP vector **(C)** and co-stained with the plasma-membrane dye, FM4-64. EGFP-mediated fluorescence derived from BdCOPT3-EGFP (COPT3) or BdCOPT4-EGFP, (COPT4), or from EGFP of the SAT6-N1-EGFP vector (EGFP) was detected using the FITC filter set while FM4-64 (FM4-64) and chlorophyll autofluorescence (Chl) were visualized using the Rhodamine filter set of an Axio Imager M2 microscope equipped with a motorized Z-drive (Zeiss). Images collected from FITC and Rhodamine filter sets were overlaid (Overlay) to show the plasma membrane subcellular localization of Cu transporters. Scale bar = 10 μm.

We next analyzed the subcellular localization of BdCOPT3 and BdCOPT4 in brachypodium using transient expression in protoplasts. After establishing a procedure for the isolation of viable protoplasts from brachypodium (Figure 6), we transfected protoplasts with BdCOPT3-EGFP or BdCOPT4-EGFP constructs with C-terminal EGFP fusions expressed from the CaMV promoter of the SAT-N1-EGFP-Gate vector, or with the empty SAT-N1-EGFP-Gate vector. We found that EGFP-mediated fluorescence originating from BdCOPT3-EGFP or BdCOPT4-EGFP constructs was located at the periphery of transfected protoplasts and did not overlap with chlorophyll-mediated autofluorescence (Figures 7A,B and Supplemental Figures 3A,B). Furthermore, fluorescence from BdCOPT3- and BdCOPT4-EGFP constructs was distinct from the fluorescence pattern exhibited by protoplasts transfected with the empty EGFP vector (Figure 7C and Supplemental Figure 3C). We note that internally-localized EGFP-mediated fluorescence is likely an artifact of the degradation of the BdCOPT-EGFPs. We then co-stained protoplasts expressing BdCOPT3- or BdCOPT4-EGFP with the lipophylic dye, FM4-64, which selectively labels the plasma membrane under low-temperature conditions (Vida and Emr, 1995). We found strict co-localization of BdCOPT3- or BdCOPT4-EGFP and FM4-64-mediated fluorescence (Figures 7A,B), suggesting that BdCOPT3 and BdCOPT4 are located at the plasma membrane.

### BdCOPT proteins interact with each other in yeast-two-hybrid assays

Homo- and heterodimerization of CTR/COPT proteins have been demonstrated in eukaryotes (Lee et al., 2002; De Feo et al., 2007, 2009; Yuan et al., 2010, 2011). Furthermore, although interaction of *A. thaliana* COPT6 with COPT1 is not required for the ability of these proteins to transport Cu (Jung et al., 2012), the activity of *O. sativa* CTR/COPT proteins seem to depend on their interactions with each other (Yuan et al., 2011). Therefore, we tested if the CTR/COPT family members in brachypodium would also interact with either themselves or/and with other family members. For this purpose we used the split-ubiquitin-based membrane yeast-two-hybrid (MYTH) approach (Kittanakom et al., 2009). In this system, a modified ubiquitin protein is split into its C- and N-terminal halves (Cub and NubG, respectively), which are fused to membrane-localized bait or prey proteins, respectively. The C-terminus of ubiquitin is attached to an artifical transcription factor, PLV (protein A-LexA-VP16). If bait and prey proteins are oriented in the cytosol and interactions occur, the modified ubiquitin is reconstituted and recognized by ubiquitin-specific proteases, which release PLV from Cub. PLV enters the nucleus to induce expression of *lexA-controlled* reporter genes *ADE2*, *HIS3*, and *lacZ* (Kittanakom et al., 2009), allowing protein interactions to be assessed by adenine and histidine prototrophy and by the β-galactosidase assay.

**Figure 8:**
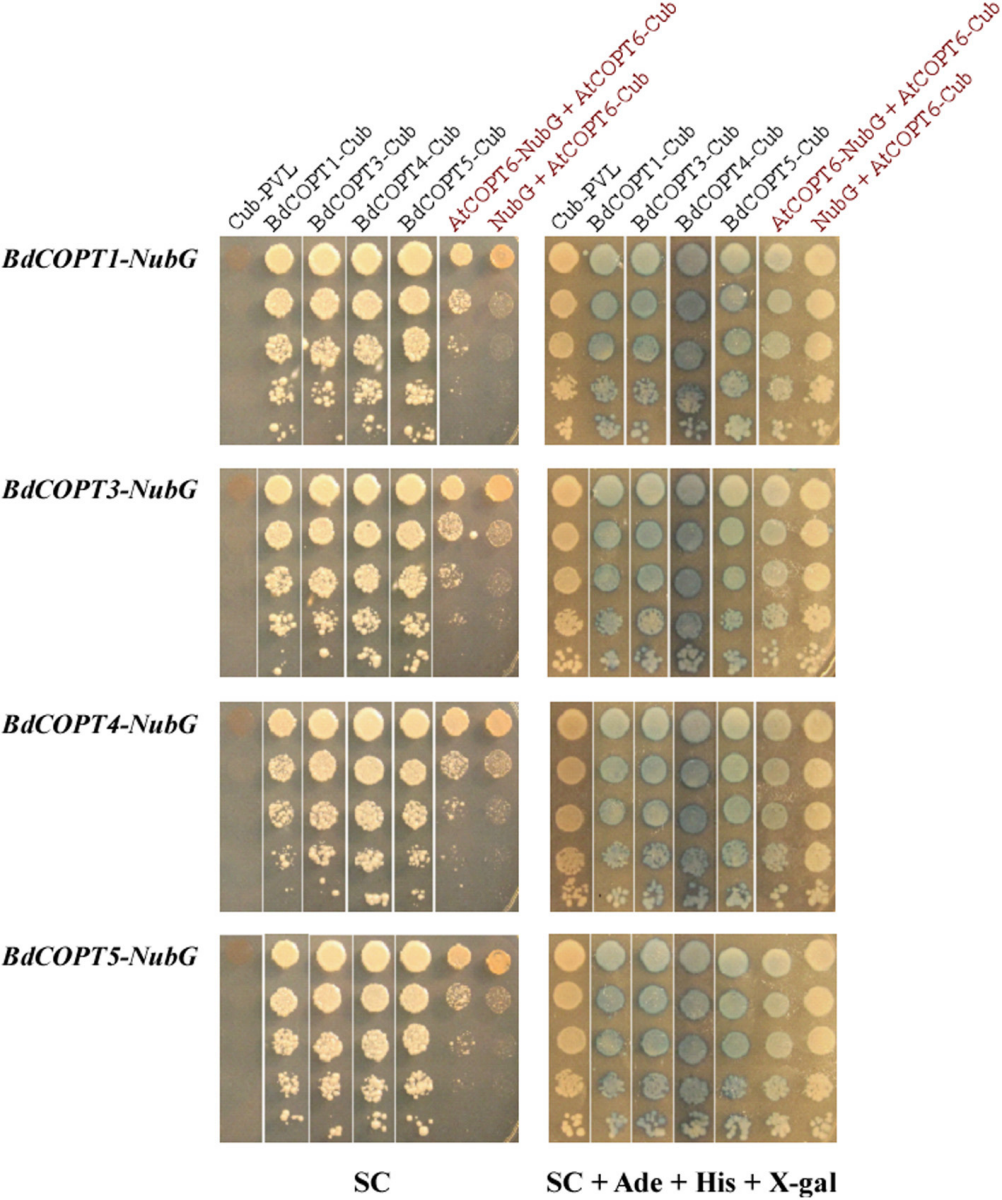
**Analyses of protein-protein interactions of brachypodium CTR/COPT transporters using the split-ubiquitin membrane yeast two-hybrid system (MYTH)**. Shown are yeast cells co-expressing NubG constructs fused with *BdCOPT1*, *BdCOPT3*, *BdCOPT4*, or *BdCOPT5* cDNA (*BdCOPT1-, BdCOPT3-, BdCOPT4-, BdCOPT5-NubG*) and a Cub-PLV construct lacking a cDNA insert (*Cub-PLV*) or Cub-PLV fused to *BdCOPT1*-, *BdCOPT3*-, *BdCOPT4*-, *BdCOPT5* (*BdCOPT1-, BdCOPT3-, BdCOPT4-, BdCOPT5-Cub*). Interactions were visualized by colony formation on selective media SC medium (SC) or as blue colonies in a ß-galactosidase assay (SC + Ade + His + X-gal). Growth was monitored for 2 days under conditions indicated below each panel. Interactions of AtCOPT6 with itself (*AtCOPT6-NubG* + *AtCOPT6-Cub*), or lack of interactions between *AtCOPT6-Cub-PLV* and the empty NubG vector (*NubG* + *AtCOPT6-Cub*) were used as controls and are indicated in red. Shown are representative results of at least three biological replicates. SC, synthetic complete medium; Ade, adenine; His, histidine; X-gal, bromo-chloro-indolylgalactopyranoside.

We fused Cub-PLV and NubG at the C-terminus of BdCOPT1, BdCOPT3, BdCOPT4, and BdCOPT5 since their predicted topology is consistent with a cytosolic orientation of their C-termini. We then co-expressed BdCOPT proteins in different combinations with themselves or with the empty Cub-PLV vector as a negative control (Figure 8). We included *S. cerevisiae* co-expressing AtCOPT6-Cub-PLV and AtCOPT6-NubG in our assays as a positive control since AtCOPT6 interacts with itself in the MYTH assay (Jung et al., 2012). To control for false positives, the empty Cub-PLV vector was co-expressed with BdCOPTs fused to NubG. These studies showed that all of the tested BdCOPT proteins interacted in both homo- and heterodimer combinations, regardless of whether interactions were assayed by growth on selective medium or with β-galactosidase (Figure 8). Whether these interactions are required for their ability to transport Cu remains to be elucidated.

## Discussion

Biological attributes of brachypodium, such as root architecture, grain structure, and the continued development of molecular and genetic resources prompted us to establish this plant as a preferred model for Cu homeostasis studies in grain cereals (e.g., wheat, barley, and oat) which are reported to be more sensitive to Cu availability in agricultural soils in comparison to other crops (Shorrocks and Alloway, 1988; Solberg et al., 1999). To begin investigations of the underlying molecular basis of this phenomenon, we initiated studies of the brachypodium CTR/COPT Cu transporters since members of this family in *A. thaliana* provide an entry point for Cu into the root and are suggested to contribute to subsequent Cu partitioning in photosynthetic tissues (Burkhead et al., 2009; Merchant, 2010; Ravet and Pilon, 2013). Toward this goal, we have identified five putative CTR/COPT family members in brachypodium based on amino acid similarity and motifs organization of CTR/COPT proteins in different species and we classified them as BdCOPT1 through 5. Phylogenetic analyses of the predicted CTR/COPT members in brachypodium and their counterparts from a dicot, *A. thaliana*, and a monocot, *O. sativa*, show that, with the exception of BdCOPT4, brachypodium and *O. sativa* CTR/COPT proteins are more closely related to each other than to *A. thaliana* (Figure 1B), reflecting a closer evolutionary relationship of brachypodium to *O. sativa* than to *A. thaliana*. Furthermore, analysis of membrane topology and the motif organization in brachypodium CTR/COPT proteins revealed that while polypeptides of all BdCOPT included the N-terminal Mets motifs, the location of M*XXX*M*-X*_12_-G*XXX*G motifs varies within different BdCOPT polypeptides, affecting the location of the positionally conserved Met residue, which is essential for the translocation of Cu across lipid bilayer (Figure 2). For example, M*XXX*M motifs are predicted to be located within TM2 only in BdCOPT3, BdCOPT4, and BdCOPT5, similar to CTR/COPT family members in other species. In contrast, M*XXX*M motifs are predicted to be located outside of TM2 in BdCOPT1 and BdCOPT2, suggesting that these proteins might not be able to mediate Cu transport due to a shift in the position of the essential Mets motifs. Although the topology predictions have to be validated experimentally, the ability of BdCOPT3, BdCOPT4, and BdCOPT5, but not BdCOPT1, to rescue partially the Cu-deficiency associated respiratory defects of the *S. cerevisiae ctr1*Δ*ctr2*Δ*ctr3*Δ mutant (Figure 4) was consistent with this suggestion. We also note that BdCOPT3, BdCOPT4, and BdCOPT5 conferred growth to the yeast mutant only when a low concentration of Cu was added to the growth medium (Figure 4). This result might be interpreted using at least two not mutually exclusive scenarios: (1) BdCOPT3, BdCOPT4, and BdCOPT5 are low affinity Cu transporters, unlike their high-affinity counterparts from *A. thaliana* and/or (2) in order to confer high affinity transport, BdCOPT3, BdCOPT4, and/or BdCOPT5 must interact with other CTR/COPTs and/or other transporters. In this regard it has been shown that most of CTR/COPTs in *O. sativa* form dimers/trimers with other CTR/COPTs or other proteins to function as high-affinity transporters (Yuan et al., 2010, 2011). In contrast, the plasma membrane-localized CTR/COPT family members from *A. thaliana* are high-affinity transporters by themselves even though can form hetero-complexes (Nakagawa et al., 2010; Jung et al., 2012; Gayomba et al., 2013). We found that brachypodium CTR/COPT transporters homo- and heterooligomerize in the MYTH system (Figure 8); whether these interactions are required for transport capabilities of brachypodium CTR/COPTs has yet to be determined.

We have also examined the subcellular localization of BdCOPTs by analyzing the localization pattern of functional BdCOPT3-EGFP and BdCOPT4-EGFP constructs by heterologous expression in yeast or transient expression in brachypodium protoplasts. Toward this goal we have established procedures for preparing viable protoplasts from brachypodium mesophyll cells and for protoplast transfection (Figure 6). These studies showed that both, BdCOPT3-EGFP and BdCOPT4-EGFP, localize to the plasma membrane, regardless of whether assays were done in yeast or protoplasts (Figures 5, 7), further suggesting that these two CTR/COPT proteins may function in Cu uptake. Finally, BdCOPT3 and BdCOPT4 genes were highly expressed in roots, and old and young leaves, and their expression was tightly regulated by Cu availability (Figure 3), as was shown for other CTR/COPT proteins from different species, including plants.

To conclude, this manuscript shows that brachypodium can be used for analyses of the molecular mechanisms underlying the increased susceptibility of small grain cereals to Cu deficiency and of Cu homeostasis overall. Analyses of the phylogenetic relationship between CTR/COPT proteins of brachypodium, *O. sativa*, and *A. thaliana*, and the clear divergence of *A. thaliana* CTR/COPT members, suggest that differences may exist in characteristics of the CTR/COPT proteins between monocots and dicots. This is further validated by studies of Cu transport capabilities of brachypodium CTR/COPTs. Heterologous expression of brachypodium CTR/COPTs in the *S. cerevisiae* Cu uptake mutant *ctr1*Δ*ctr2*Δ*ctr3*Δ suggest that increased sensitivity to Cu deficiency in some grass species may arise from lower efficiency of Cu uptake and, possibly, other properties of components of Cu uptake and tissue partitioning systems. It is also possible that the CTR/COPT family members in brachypodium require homo- or heterooligomerization for high-affinity Cu uptake, unlike corresponding family members in *A. thaliana*, reinforcing the importance of using brachypodium as a model for the comprehensive analyses of Cu homeostasis in cereal crops.

### Conflict of interest statement

The authors declare that the research was conducted in the absence of any commercial or financial relationships that could be construed as a potential conflict of interest.

## Supplementary material

Supplemental Figure 1 **Establishing growth conditions for brachypodium**. Plants were grown on standard nutrient solution at 22°C and 12-h light/12-h dark photoperiod at photosynthetic photon flux density of 150 μmol photons m^−2^ s^−1^. The glume and lemma of seeds were removed and seeds were sown on rinsed perlite **(A)**. Seven-day-old seedlings grown on perlite irrigated with standard nutrient solution **(B)**. Seven-day-old seedlings were transferred from perlite to hydroponic medium and shown from above **(C)** and from the side view **(D)**. Scale bar = 5 cm.

Supplemental Figure 2 **Phenotypes of brachypodium grown under different copper conditions. (A)** Twenty five-day-old plants grown in hydroponic solution without CuSO_4_ and supplemented with 500 μM BCS (*500 BCS*), or indicated concentrations of CuSO_4_. **(B)** Plants from **(A)** were used to measure height (*Plant height*), shoot dry weight (*Shoot dry weight*), and root dry weight (*Root dry weight*). Asterisks (^**^) indicate statistically significant differences (*p* ≤ 0.01, ANOVA) of the mean values of treatments compared to control conditions (0.25 μM CuSO_4_). Error bars show *SD* (*n* = 3).

Supplemental Figure 3 **Subcellular localization of BdCOPT3 and BdCOPT4 in brachypodium protoplasts**. Protoplasts were transfected with BdCOPT3-EGFP **(A)** or BdCOPT4-EGFP **(B)** constructs or empty SAT6-N1-EGFP vector **(C)** and co-stained with the plasma-membrane dye, FM4-64. EGFP-mediated fluorescence derived from BdCOPT3-EGFP (COPT3) or BdCOPT4-EGFP, (COPT4), or from EGFP of the SAT6-N1-EGFP vector (EGFP) was detected using the FITC filter set while FM4-64 (FM4-64) and chlorophyll autofluorescence (Chl) were visualized using the Rhodamine filter set of an Axio Imager M2 microscope equipped with the motorized Z-drive (Zeiss). Images collected from FITC and Rhodamine filter sets were overlaid (Overlay) to show the plasma membrane subcellular localization of the Cu transporters.

Supplemental Table 1 **Primers used in this study**. Sequences in italics indicate *att* sites used for Gateway cloning and “^*^” indicate sequences without a stop codon.

Supplemental Table 2 **Solutions used in protoplast isolation and transfection**. All solutions were filter sterilized before use.
