# Supplementary figures and images for: Brachypodium distachyon as a model system for studies of copper transport in cereal crops
Source: Front Plant Sci. 2014 May 30;5:236. doi: 10.3389/fpls.2014.00236 (PMC4039008; doi:10.3389/fpls.2014.00236)

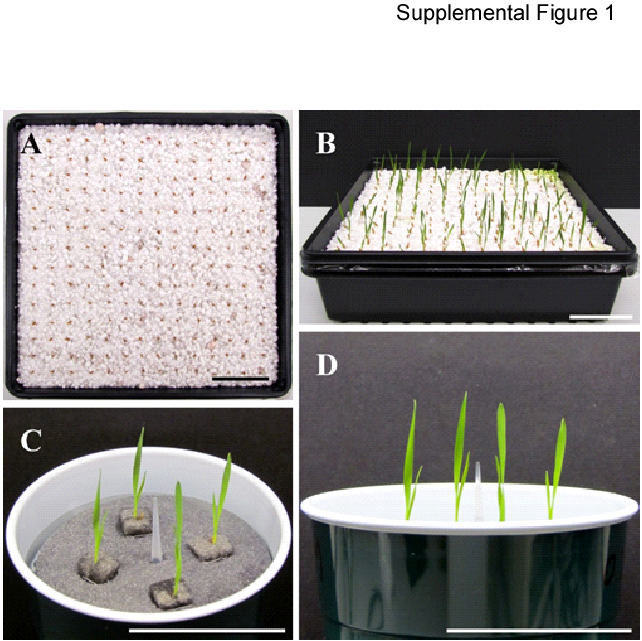

Supplement: Supplemental Figure 1 — Establishing growth conditions for brachypodium. Plants were grown on standard nutrient solution at 22°C and 12-h light/12-h dark photoperiod at photosynthetic photon flux density of 150 μmol photons m−2 s−1. The glume and lemma of seeds were removed and seeds were sown on rinsed perlite (A). Seven-day-old seedlings grown on perlite irrigated with standard nutrient solution (B). Seven-day-old seedlings were transferred from perlite to hydroponic medium and shown from above (C) and from the side view (D). Scale bar = 5 cm. [file Presentation1.ZIP › 85981_Sup Fig 1.TIF]

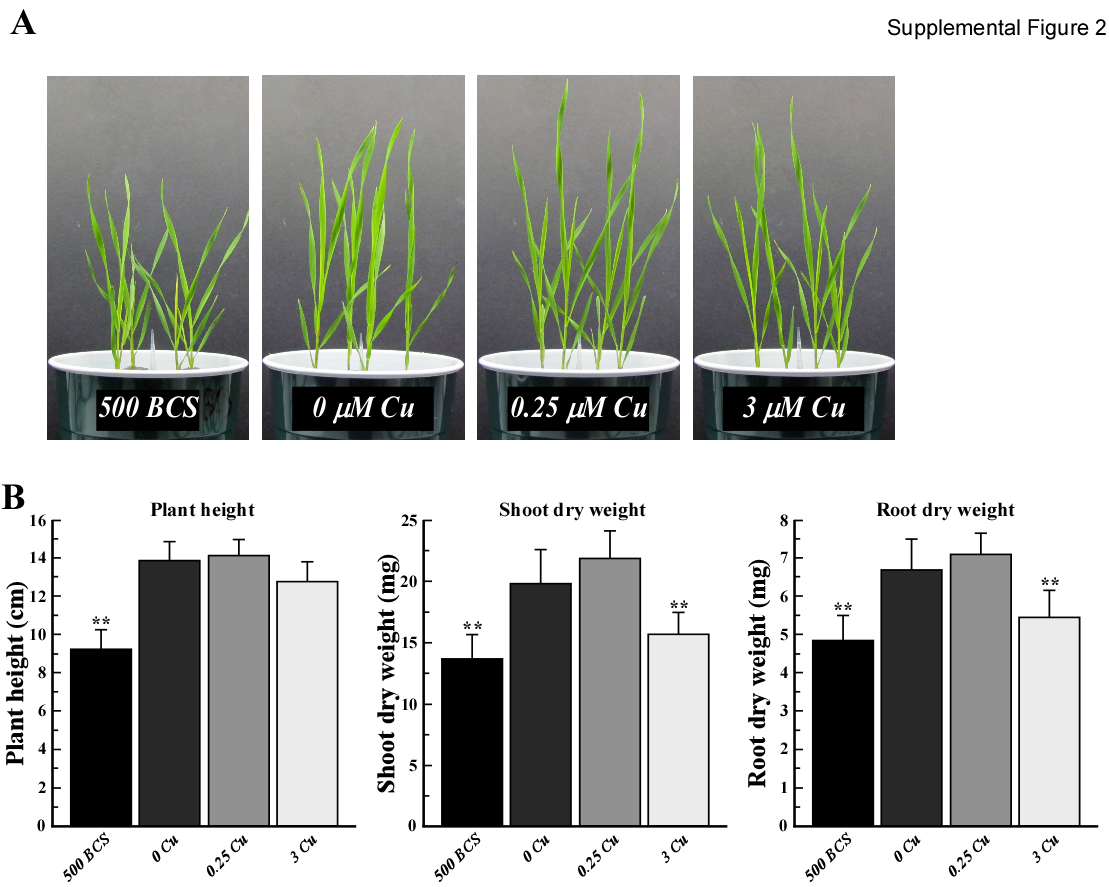

Supplement: Supplemental Figure 1 — Establishing growth conditions for brachypodium. Plants were grown on standard nutrient solution at 22°C and 12-h light/12-h dark photoperiod at photosynthetic photon flux density of 150 μmol photons m−2 s−1. The glume and lemma of seeds were removed and seeds were sown on rinsed perlite (A). Seven-day-old seedlings grown on perlite irrigated with standard nutrient solution (B). Seven-day-old seedlings were transferred from perlite to hydroponic medium and shown from above (C) and from the side view (D). Scale bar = 5 cm. [file Presentation1.ZIP › 85981_Sup Fig 2.TIF]

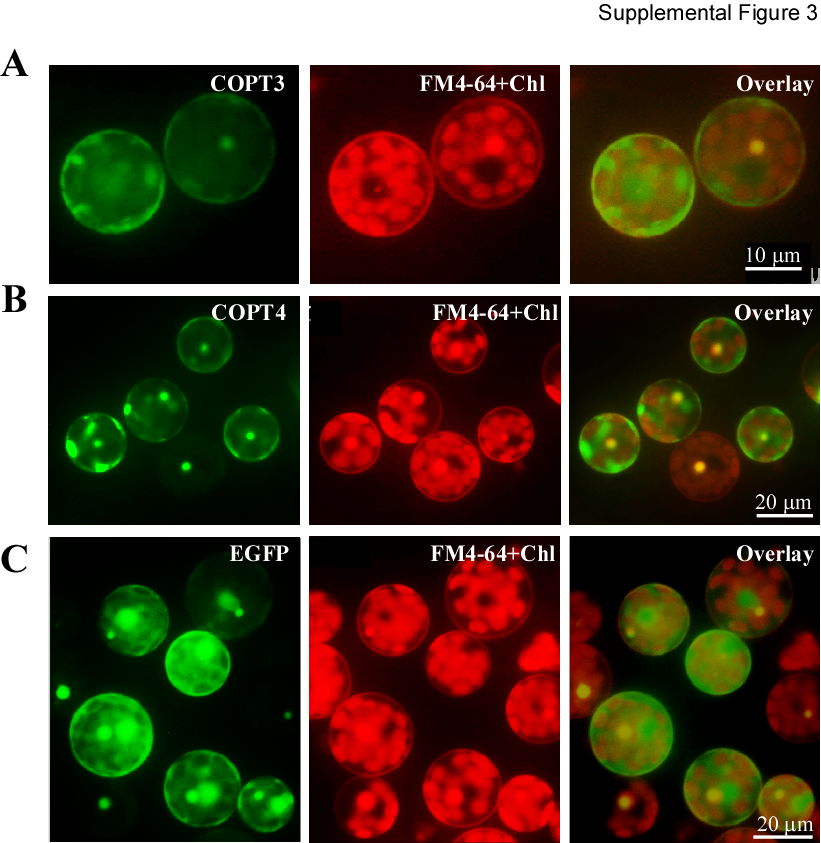

Supplement: Supplemental Figure 1 — Establishing growth conditions for brachypodium. Plants were grown on standard nutrient solution at 22°C and 12-h light/12-h dark photoperiod at photosynthetic photon flux density of 150 μmol photons m−2 s−1. The glume and lemma of seeds were removed and seeds were sown on rinsed perlite (A). Seven-day-old seedlings grown on perlite irrigated with standard nutrient solution (B). Seven-day-old seedlings were transferred from perlite to hydroponic medium and shown from above (C) and from the side view (D). Scale bar = 5 cm. [file Presentation1.ZIP › 85981_Sup Fig 3.TIF]
