# Supplementary material for: Brachypodium distachyon as a model system for studies of copper transport in cereal crops
Source: Front Plant Sci. 2014 May 30;5:236. doi: 10.3389/fpls.2014.00236 (PMC4039008; doi:10.3389/fpls.2014.00236)
Supplement: Supplemental Table 1 — Primers used in this study. Sequences in italics indicate att sites used for Gateway cloning and “*” indicate sequences without a stop codon. [file Presentation1.PDF]

**Supplemental Table 1.** Primers used in this study. Sequences in italics indicate *att* sites used for Gateway cloning and * indicate sequences without a stop codon.

| **Gene** | **5´ → 3´** | **Purpose** |
|---|---|---|
| *BdCOPT1-F* | atggccatgccgatgccgatg | qPCR |
| *BdCOPT1-R* | atgcccatgtccatgtccatccct | qPCR |
| *BdCOPT2-F* | ccggagcagcgtaaacttgaca | qPCR |
| *BdCOPT2-R* | ggcatcggcatggacatcttgttt | qPCR |
| *BdCOPT3-F* | atggacatgggaggagggcatca | qPCR |
| *BdCOPT3-R* | aaggtcatgtgcatgtagtgcgtc | qPCR |
| *BdCOPT4-F* | tcatgctcgccatcatgtcgtt | qPCR |
| *BdCOPT4-R* | ttgtcgaccacgacgagatcct | qPCR |
| *BdCOPT5-F* | tatctcgtgatgctggcggtcat | qPCR |
| *BdCOPT5-R* | tacggcttggatccgttcgact | qPCR |
| *BdActin-F* | tggattggaggatccatcttggca | qPCR |
| *BdActin-R* | agcatttcctgtgcacaatggacg | qPCR |
| *BdCOPT1-F* | *ggggacaagtttgtacaaaaaagcaggcttc*atggccatgccgatgccgat | Gateway cloning |
| *BdCOPT1-R* | *ggggaccactttgtacaagaaagctgggtc*ctaaggtttgggctcagctgcct | Gateway cloning |
| *BdCOPT1-R** | *ggggaccactttgtacaagaaagctgggtc*aggtttgggctcagctgcct | Gateway cloning |
| *BdCOPT3-F* | *ggggacaagtttgtacaaaaaagcaggcttc*atggacatgggaggagggcat | Gateway cloning |
| *BdCOPT3-R* | *ggggaccactttgtacaagaaagctgggtc*ctagcagcacgccgcc | Gateway cloning |
| *BdCOPT3-R** | *ggggaccactttgtacaagaaagctgggtc*gcagcacgccgccggc | Gateway cloning |
| *BdCOPT4-F* | *ggggacaagtttgtacaaaaaagcaggcttc*atgatgcacatgaccttctactgg | Gateway cloning |
| *BdCOPT4-R* | *ggggaccactttgtacaagaaagctgggtc*ctacgcgcaggcgca | Gateway cloning |
| *BdCOPT4-R** | *ggggaccactttgtacaagaaagctgggtc*cgcgcaggcgcag | Gateway cloning |
| *BdCOPT5-F* | *ggggacaagtttgtacaaaaaagcaggcttc*atggcgccgccgcgg | Gateway cloning |
| *BdCOPT5-R* | *ggggaccactttgtacaagaaagctgggtc*ctacggcttggatccgttcgactgc | Gateway cloning |
| *BdCOPT5-R** | *ggggaccactttgtacaagaaagctgggtc*cggcttggatccgttcgactgc | Gateway cloning |

**Supplemental Table 2.** Solutions used in protoplast isolation and transfection. All solutions were filter sterilized before use.

| Name | Composition |
|---|---|
| TVL solution | 0.3 M sorbitol, 50 mM $CaCl_2$ |
| Enzyme solution | 0.5 M sucrose, 20 mM $CaCl_2$, 40 mM KCl, 1.5% (w/v) Cellulase, 1% (w/v) Macerozyme, 20 mM MES-KOH, pH 5.7 |
| W5 solution | 0.037% (w/v) KCl, 0.9% (w/v) NaCl, 1.84% (w/v) $CaCl_2$, 2 mM MES-KOH, pH 5.7 |
| MMG solution | 0.4 M mannitol, 15 mM $MgCl_2$, 4 mM MES-KOH, pH 5.7 |
| PEG-Calcium solution | 0.2 M mannitol, 100 mM $CaCl_2$, 40% PEG-4000 |
